# Protein Kinases at the Intersection of Translation and Virulence

**DOI:** 10.3389/fcimb.2019.00318

**Published:** 2019-09-11

**Authors:** Jay Leipheimer, Amanda L. M. Bloom, John C. Panepinto

**Affiliations:** Department of Microbiology and Immunology, Jacobs School of Medicine and Biomedical Sciences, University at Buffalo, Buffalo, NY, United States

**Keywords:** translation, kinase, fungi, translational regulation, stress response, oxidative stress, heat shock, starvation adaptation

## Abstract

As free living organisms, fungi are challenged with a variety of environmental insults that threaten their cellular processes. In some cases, these challenges mimic conditions present within mammals, resulting in the accidental selection of virulence factors over evolutionary time. Be it within a host or the soil, fungi must contend with environmental challenges through the production of stress effector proteins while maintaining factors required for viability in any condition. Initiation and upkeep of this balancing act is mainly under the control of kinases that affect the propensity and selectivity of protein translation. This review will focus on kinases in pathogenic fungi that facilitate a virulence phenotype through translational control.

## Introduction

Survival in a mammalian host ultimately requires the invading fungus to combat three main physiological challenges; (1) It must be able to acquire nutrients in a restricted resource environment; (2) withstand temperatures exceeding ambient, and (3) contend with immune system-induced killing in the form of acidic and oxidative damage. These restrictions may be the very reason that of the over 2 million predicted fungal species, only ~300 have ever been found to cause disease (O'brien et al., 2005). Therefore, possession and production of factors that allow an organism to remain viable under these conditions are necessary for mammalian virulence. So what do these organisms possess that allows them to overcome these challenges? Genomic sequencing has failed to adequately address this question as species within the same genus vary in their pathogenicity. For example, *Cryptococcus amylolentus* is not pathogenic despite having most of the virulence factors possessed by its highly virulent relative *Cryptococcus neoformans* (Garcia-Solache et al., 2013).

We speculate that an organism's pathogenic potential can be defined by its “adaptive agility” to host derived cellular stress, that is the *extent* and *speed* at which an organism can reshape its proteome to one that is suited for a new environment. The *extent*, that is the maintenance and longevity of a stress response, is partially controlled at the level of transcriptional expression through the sustained production of responsive genes. However, the *speed* at which the response is executed is determined by cellular factors already present at the time of the stress and are made following it to amplify the transcriptional response. At first glance, one would not believe that the *speed* of the stress response would be necessary for fungi to promote infection. However, many of the host derived cellular insults have immediate deleterious effects that must be contented with immediately before the organism sustains irreparable damage. Rapid expression of virulence factors in response to quickly changing environmental conditions could alter the outcome of infection toward progression rather than containment and clearance. Therefore, the interaction between the host and pathogen at the molecular level can be viewed as a competition between organisms' “adaptive agilities,” where a host response is met with a pathogenic response and vice versa.

The biological exchange can be played out over time (chronic infection) or can be decided quickly if one of the opponents is faster than the other (acute infection/death). An example of this competitive dynamic can be seen between the dimorphic fungi *Candida albicans* and the phagocytosing macrophage (Vazquez-Torres and Balish, 1997; Klengel et al., 2005; Brothers et al., 2011). In response to signals such as increasing levels of CO_2_ and reactive oxygen species (ROS), *C. albicans* responds by filamenting to prevent phagocytosis or to escape the macrophage if already phagocytosed. As the pathogen initiates the process of germination, the macrophage itself upregulates genes involved in phagosome maturation (Nicola et al., 2008). Genetic manipulations that slow down the maturation process in the macrophage result in failed fungal killing (Okai et al., 2015), whereas those that cause a defect in filamentation result in macrophage induced killing (Ghosh et al., 2009). A draw is met when both competitors reach homeostasis and limit their responses (latency). Low transcriptional output is a characteristic of fungal latency, with most activity involving nutrient acquisition and autophagy instead of growth as seen with an active infection (Alanio et al., 2015; Brunet et al., 2018). An example of immune latency is best characterized in response to *Mycobacterium tuberculosis*, where macrophages continually fail to clear the pathogen and instead aggregate around the site of infection forming a granuloma (Flynn and Chan, 2001; Pagan and Ramakrishnan, 2014).

How an organism responds to an environmental insult produced by the host is determined by the coordinated efforts of multiple cellular processes. The disruption of a single signaling pathway resulting in the loss of many virulence traits supports this claim (Zhao et al., 2006; Hu et al., 2008b; Lee et al., 2016). One of the most immediate and drastic effects that can occur in response to cellular stress are changes in the translational landscape of the organism, which in most cases precedes the transcriptional response. Many of these changes that influence the translational state are brought about by post-translational modifications in the form of protein phosphorylation. Therefore, kinases have the potential to manipulate the *speed* of a stress response by modulating the association of individual mRNAs with translation related factors in response to cellular stress. Furthermore, translation can control the *extent* of the stress response by limiting the number of active and available ribosomes at the time of stress. Initiation is generally considered the rate-limiting step in protein synthesis, making it a prime regulatory point for controlling translation output.

This synopsis aims to discuss the role kinases play in regulating translation in response to and during host derived stress. In particular, we will focus on kinase-mediated regulation of translation initiation brought about by exposure to ROS (oxidative stress), higher temperatures, and the demand for alternative carbon utilization for energy production. Kinase mediated regulation of translation is critical for rapidly promoting the expression of factors that allow for survival in the host and therefore, virulence.

## An Overview of Translation and Its Points of Regulation

**Figure 1:**
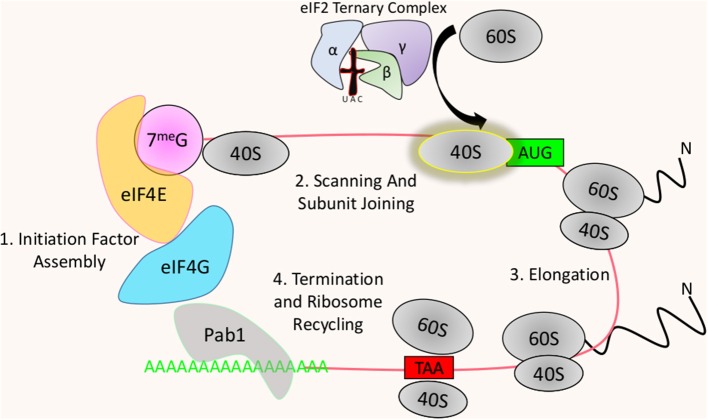
Brief overview of translation: (1) Translation begins with the recruitment of the cap-binding complex to the 5′ end of the mRNA allowing interaction with the Poly-A binding protein (Pab1)-associated 3′ poly-A tail. All Eukaryotic mRNAs are capped by a modified nucleotide at the 5′ end, which is typically a N7-methylated guanosine. eIF4E recognizes the cap and recruits the scaffolding initiation factor eIF4G. eIF4G itself recruits a suite of initiation factors including Pab1, which binds to the poly-a tail. The interaction between eIF4G and Pab1 bridges the 5′ and 3′ ends of the mRNA forming a “closed loop” structure that is thought to stimulate translation. (2) At this point the 40S subunit is recruited to the 5′ end of the mRNA where it begins scanning the transcript until reaching a start codon. The corresponding initiator tRNA is delivered to the 40S subunit at the start site by the eIF2 ternary complex, allowing for the joining of the 60S subunit (3). Following ribosome assembly, translation elongation begins with the recruitment of tRNAs charged with their respective amino acids. (4) Elongation ends when the ribosome recognizes a stop codon, where no corresponding tRNA exists, resulting in the release of the produced polypeptide and the dissociation of the 60S and 40S subunits thereby allowing their use for further rounds of translation.

The process of translation in eukaryotic cells involves several specific stepwise associations of factors with mRNAs, followed by a cascade of events that lead to translation initiation. The ribosome then synthesizes the polypeptide through the process of translation elongation, eventually terminating upon reaching a stop codon (Figure 1). This process was once believed to be unregulated with most gene expression being controlled solely at the transcriptional level. However, strong evidence now suggests that multiple steps of mRNA decoding are heavily modulated under select conditions, with imbalances having drastic consequences to cellular functions (Le Quesne et al., 2010; Spriggs et al., 2010; Genuth and Barna, 2018). Within this cycle of protein synthesis, several regulatory checkpoints are targeted by kinases to exert regulatory control of the process. Initiation is the least evolutionarily conserved process of translation and is also believed to be the rate-limiting step (Benelli et al., 2003).

Eukaryotic small ribosomal subunits cannot interact directly with mRNAs, with a few rare exceptions. Instead, translation initiation involves the recruitment of the 40S pre-initiation complex to the 5′ methylated cap of the mRNA (Gross et al., 2003). This is achieved through the cooperating efforts of the cap binding complex with the direct cap binding protein 4E, recruiting the scaffolding protein 4G, and the helicase 4A that unwinds mRNA secondary structure between the cap and the start codon during ribosomal scanning (Gross et al., 2003; Schutz et al., 2008; Marintchev et al., 2009) (Figure 1). Most translationally active mRNAs contain tracts of adenines at their 3′ end called a poly-A tail, which can be bound by the Poly-A binding protein (Pab1) (Blobel, 1973). Pab1 is believed to associate with 4G, circularizing the transcript forming what is known as a “closed loop” where the 3′ tail is brought close to the 5′ cap (Sachs and Davis, 1989; Wells et al., 1998). It is important to note, however, that this association is not strictly required for mRNA translation but instead may enhance it (Thompson and Gilbert, 2017). Joining of the 60S subunit with the 40S subunit at the start codon so that that protein biogenesis can begin, is accomplished through the recruitment of the initiator tRNA bound to the ternary complex (Liemburg-Apers et al., 2015). Following the recruitment of the 40S pre-initiation complex to the 5′ cap of the mRNA and subsequent scanning along the 5′ UTR (Untranslated Region), protein biogenesis begins with the joining of the 60S subunit with the ternary complex containing the initiator tRNA. The ternary complex, which is composed of 3 subunits (eIF2α, β, γ), hydrolyzes bound GTP to GDP after delivering the initiator tRNA to the peptidyl site (P-Site) of the ribosome (Jackson et al., 2010). Recycling of this factor requires the exchange of the now bound GDP with GTP.

The rate of initiation, and by extension, the rate of translation, can be modulated by interfering with the cooperative binding efforts between these factors. Furthermore, inhibition of proteins that recognize the 5′ cap or the 3′ poly-A tail will also limit or prevent translation. The stress conditions mentioned in this review modulate kinase activity to directly or indirectly affect these dynamic interactions, which result in a change in the efficiency and specificity of mRNA translation. Each fungal stress response can be characterized by the combinatory action of kinases that distinctly change the post-translational make-up and overall concentration of translation related factors. We will discuss results that suggest that these stress induced changes made by kinases on the translational machinery act to improve the “adaptive agility” of the pathogen.

## Kinases Controlling Translation in Response to Oxidative Stress

Fungal pathogens that can penetrate a host's skin or mucosal layer are first met with innate immune cells. *Cryptococcus neoformans* and *Pneumocystis spp*. are cleared primarily by macrophages, whereas neutrophils are more critical in clearing *Candida albicans* and *Aspergillus fumigatus*, depending upon their morphotypic state (Vazquez-Torres and Balish, 1997; Dunyak et al., 2002; Nicola et al., 2008; Bhagwat et al., 2018). Macrophages internalize yeast through the process of phagocytosis followed by the intracellular production of reactive oxygen species (ROS) and reactive nitrogen species (RNS), which are potent fungicidal effector molecules (Nicola et al., 2008). Filamentous fungi, however, are exposed to extracellular forms of ROS produced via neutrophils (Bonnett et al., 2006). Regardless of its form or location, ROS will produce fungicidal effects unless the organism can quickly reduce its cellular components and repair the damage caused by oxidization (Morano et al., 2012). Transcriptome analysis of *C. albicans* exposed to macrophages suggests that phagocytosis elicits a transcriptional response in which mRNAs encoding proteins related to translation are repressed, while those related to countering oxidative stress and alternative carbon utilization pathways are upregulated (Lorenz et al., 2004). *C. neoformans* and *A. fumigatus* have a similar pattern of expression when exposed to macrophages and neutrophils, respectively (Sugui et al., 2008; Derengowski Lda et al., 2013). Together, these results suggest that fungi respond to oxidative damage by downregulating biological processes involved in growth and ribosome biogenesis and instead place higher importance on the production of factors related to ROS reduction and cellular damage repair. How fungi can initiate such a drastic shift in phenotypic expression may involve intense regulation of active ribosomes (Mills and Green, 2017).

### eIF2α Phosphorylation Mediated Translational Regulation

**Figure 2:**
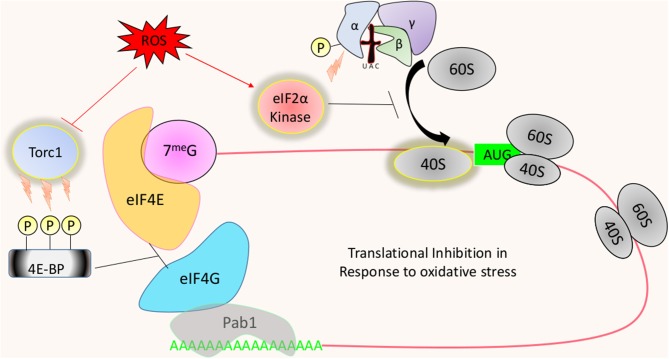
Translational regulation in response to oxidative stress: ROS will lead to the activation of eIF2α kinases, such as Gcn2, to inhibit the recycling of the ternary complex. This results in the overall reduction of protein synthesis, with certain mRNAs either translationally favored or resistant to the suppression. Translation can also be inhibited through the de-activation or Tor1 in response to oxidative stress. Tor1 phosphorylates 4E-BP (Eap1 or Caf20 in yeast) to prevent it from inhibiting the eIF4E and eIF4G interaction, which acts to increase the rate of ribosome recruitment.

Technical limitations remain in studying translational responses to dynamic interactions such as those found between host and pathogen. Manipulation of culture media, however, can mimic a stress environment that a fungus encounters in the host. One such method is the utilization of ROS generating compounds such as hydrogen peroxide (H_2_O_2_). Experiments such as these have revealed dramatic changes to the translational landscape in yeast, with translation initiation receiving the strongest regulatory pressure (Shenton et al., 2006). However, it is important to note that elongation may also be affected as well (Grant, 2011; Wu et al., 2019). The best example of translational control in response to oxidative stress are found in *S. cerevisiae*. Exponentially growing cultures exposed to hydrogen peroxide undergo global translational repression (Holmes et al., 2004). Translational repression was alleviated in the absence of the kinase Gcn2 (general control non-derepressible 2), which is the sole kinase of the eIF2α subunit in the ternary complex (Wek et al., 1995). Phosphorylation of this subunit at a single conserved serine by this serine/threonine protein kinase prevents the ternary complex from recycling new initiator tRNAs (Wek et al., 1995; Zaborske et al., 2009) (Figure 2). As a result, new rounds of canonical translation cannot continue due to the lack of available active ternary complex required for 80S subunit formation at the start codon. It is interesting to find Gcn2 possessing catalytic activity in response to H_2_O_2_, as the only known activation pathway for the kinase involves binding of uncharged tRNA to a functionally essential domain (Ramirez et al., 1992). Therefore, H_2_O_2_ and other non-nitrogen starving conditions known to induce Gcn2-dependent eIF2α phosphorylation must somehow affect the availability of amino-acylated tRNA to initiate translational inhibition (Anda et al., 2017). Rapid and tight control over the abundance of charged tRNAs may provide a level of gene regulation that occurs before other forms of translational remodeling (Hanson and Coller, 2018).

Preventing translational suppression in response to oxidative stress results in a severe growth sensitivity in *S. cerevisiae* (Shenton et al., 2006). How translational suppression facilitates the observed increase in fitness remains obscure. It is known, however, that specific mRNAs can escape translational repression and instead experience translational upregulation (Lu et al., 2004; Vattem and Wek, 2004). Regulating translation in this way can be a strategy to quickly deploy a stress response by establishing new conditions under which the rate of initiation for specific transcripts is favored over others. A classic example in support for this hypothesis is seen in the translational de-repression of the transcription factor Gcn4p, where high levels of active ternary complex favoring ribosomes to associate at start codons upstream of the annotated start codon (uORF) preventing the coding ORF from being translated (Lu et al., 2004). Gcn2 activation reduces active ternary complex, thereby, lowering the recognition of weaker start codons and instead favors those in proper Kozak context. Higher eukaryotes can possess up to 3 other distinct eIF2α kinases (except for some fish that can have 5), in addition to Gcn2, that are activated in a tRNA-independent process (Baird and Wek, 2012). Many fungal pathogens possess far fewer, with *C. albicans* and *C. neoformans* containing only one Gcn2 homolog (Tournu et al., 2005). *A. fumigatus* seems to possess an additional eIF2α kinase, possibly Heme-Regulated Inhibitor (HRI), as strains lacking the Gcn2 homolog CpcC (Cross-Pathway Control C) have equivalent levels of phosphorylated eIf2α as wild type strain in response to nitrogen starvation (Sasse et al., 2008). Growth in culture media alone is uninhibited for *S. cerevisiae* lacking Gcn2, which is in stark contrast to *A. fumigatus* and *C. albicans* in which the absence of Gcn2 results in severe growth and morphogenetic defects (Tournu et al., 2005; Sasse et al., 2008). The differing outcomes of gene deletion observed suggest that these fungi have adopted additional roles for Gcn2 outside the scope of the environmental stress response.

### Tor Mediated Translational Regulation

Oxidative stress can also lead to translational suppression by manipulating the formation of the cap-binding complex. *Saccharomyces cerevisiae* strains lacking the mammalian homolog of 4E-BP (4E binding protein), Eap1 (eIF4E-associated protein 1), display a reduced sensitivity to certain forms of oxidative stress mediated translational suppression (Figure 2). The binding of eIF4G to a specific domain on eIF4E is crucial for the canonical process of translation to begin. Un-phosphorylated Eap1 and Caf20 compete with eIF4G for the corresponding domain found on eIF4E, preventing the recruitment of 4G, which then prevents the recruitment of the 40S subunit (Altmann et al., 1997). Typically this inhibitory action is thought to be repressed through the constitutive catalytic activity of Tor1 (Target of Rapamycin 1), as protein synthesis in *eap1*Δ strains is unaffected by rapamycin treatment (Matsuo et al., 2005).

Tor1 is a phosphoinositide kinase-related protein kinase (PIKK) family of atypical Ser/Thr-specific kinases that control cell growth through targeting of multiple factors related to ribosome biogenesis and translation (Keith and Schreiber, 1995; Huber et al., 2009). Long term effects of Tor1 activity regulate multiple aspects of ribosome biogenesis, mainly at the level of transcription (Huber et al., 2009). However, its deactivation in response to stress has been found to reduce not only translation but also increase mRNA turn-over (Albig and Decker, 2001). Tor signaling has traditionally been viewed as a nutrient response pathway, but evidence now shows it to be much more versatile. As stated earlier, de-repression of Eap1 in response to specific oxidative stresses was found to be important in proper translational inhibition in *S. cerevisiae* (Mascarenhas et al., 2008). Furthermore, Tor inactivation in response to hypoxia (an inducer of ROS stress) reduces the translational efficiency of mRNA bearing a 5′ terminal oligopyrimidine (TOP) tract in their 5′ UTR (Spriggs et al., 2010). Most mRNAs containing these motifs are associated with ribosome biogenesis and translation. Tor inactivation instead creates a translational system that favors mRNAs predicted to have large structures in their 5′ UTRs. These transcripts tend to be those associated with stress responses, membrane transport, and acute signaling processes. In *C. albicans*, Tor1 was found to promote filamentous growth while negatively regulating many cell wall and adhesion genes (Bastidas et al., 2009). Changes in translational efficiency of mRNAs under rapamycin treatment have yet to be performed in any fungal pathogens, to the best of our knowledge. Ribosome Profiling, a technique that can measure the relative levels of ribosome bound mRNA over the total mRNA, may now allow researchers to address this question more easily (Ingolia et al., 2011).

## Kinase Mediated Translational Adaptation to Glucose Starvation

Fungi is a diverse kingdom made of single and multicellular saprophytes. Survival in the environment and the host requires the use of flexible carbon acquisition strategies that can respond to fluctuation in availability (Passalacqua et al., 2016). The preferred carbon source for pathogenic fungi may be limited within the host requiring the expression of a versatile repertoire of nutrient acquisition strategies for successful infection (Oliver et al., 2002). Different niches, for example, glucose-rich central nervous system vs. poor nutrient tissue, require the promotion of different carbohydrate utilization pathways (Cooney and Klein, 2008). Evidence for the demand for alternative energy acquisition is seen in both *C. neoformans* and *C. albicans* where pathways that generate energy in the absence of free glucose are required for long term survival in the host (Lorenz and Fink, 2001; Price et al., 2011). Macrophage phagocytosis also restricts invading pathogen's access to glucose. Transcriptional profiling of fungi that are taken up by macrophages shows substantial upregulation in genes related to alternative carbon source utilization and carbon transportation (Lorenz et al., 2004; Derengowski Lda et al., 2013).

**Figure 3:**
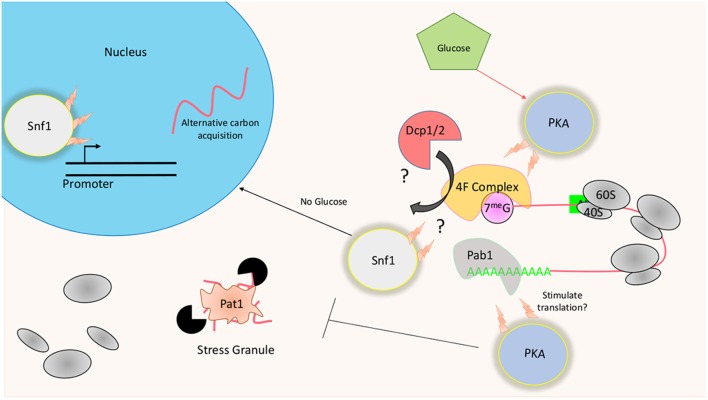
Translational regulation in response to glucose availability: The presence of glucose results in the activation of PKA promoting high levels of protein biogenesis. At the translational level, this may be achieved through the phosphorylation of Dcp1/2 or Pat1. Phosphorylation of the decapping enzymes may weaken interactions with the 5′ cap, instead allowing the translational machinery to associate with the mRNA. PKA may also act to stimulate translation through post-translational modifications of Pab1. Snf1 kinase is known to promote the transcription of the alternative carbon utilization pathway. Long term effects of its deletion may promote a proteome that is already preadapted to glucose mediated translational suppression.

Removing glucose from culturing media results in dramatic changes at both the transcriptional and translational level in yeast. Original studies using polysome profiling and radioactive methionine incorporation readouts found that translation is largely inhibited within minutes in *S. cerevisiae* following a shift to glucose-depleted conditions (Ashe et al., 2000). Furthermore, the observed repression was found to be necessary for an appropriate starvation response to be formed at the transcriptional level (Ashe et al., 2000; Arribere et al., 2011). How this repression comes about and why it has an effect on transcriptional induction is still mostly unknown. Polysome profiling performed in *S. cerevisiae* using; (1) strains lacking certain regulators of the kinase Snf1 (Sucrose NonFermenting 1); (2) particular subunits of Protein Kinase A (PKA) complex, and (3) enzymes responsible for mRNA 5′ de-capping all show a resistance to translational inhibition in response to glucose withdraw (Ashe et al., 2000; Holmes et al., 2004) (Figure 3).

### Snf1 Mediated Regulation of Translation

The transient activation of heterotrimeric protein-serine/threonine kinase Snf1 correlates with 80S ribosome disassembly following the removal of glucose (Hedbacker and Carlson, 2008). Carbon starvation induces robust phosphorylation of Snf1 in the cell, with levels slowly returning to basal over time following carbon starvation (Hedbacker and Carlson, 2008). Although commendable effort has been made to determine the mechanism through which the observed rapid reduction in translation is brought about, the exact causative means is still unknown. What seems clear is that the mechanisms of translational inhibition in response to glucose starvation are starkly different from that of other forms previously identified. For example, the phosphorylation of eIF2α or 4E-BP, which reduces active ternary complex or disrupts the cap binding complex respectfully, do not play a role in this glucose mediated suppression.

Research relating to the role of Snf1 in promoting alternative forms of carbon utilization mainly focuses on its stimulatory effect on transcription (Tu and Carlson, 1995). How it can affect translational repression is far less understood. Strains lacking Reg1 and Hxt2, which inhibit the Snf1 catalytic activity, are resistant to translational repression in response to glucose removal (Ashe et al., 2000). Ablating Snf1 in these strains restores translational repression. Given this information, uninhibited Snf1 catalytic activity in the presence of glucose may promote the formation of translational machinery that is unresponsive to carbon starvation despite not having experienced this condition. The proposed pre-adapted translational machinery could arise from either the transcriptional promotion of alternative carbon source response genes or modifications of existing translational components. Translational output in response to carbon starvation does increase several hours following the initial shut-down, although never to original levels (Vaidyanathan et al., 2014). Why translation is allowed to proceed at this time point but not earlier is unknown. However, Snf1 catalytic activity might be responsible for promoting this new translational phenotype during the initial starvation period through the modification of existing ribosome machinery and the production of new components. Accepting this hypothesis would require one to assume that the ribosome composition is dynamic, not static, with its make-up defining mRNA preference and translational potential (Genuth and Barna, 2018). Recent work quantitatively comparing ribosome associated protein during starved and fed conditions in *S. cerevisiae* lends support to this hypothesis (Wang et al., 2018).

### PKA and De-capping Mediated Regulation of Translation

Protein kinase A (PKA) is a serine/threonine kinase composed of two regulatory subunits that bind and inactivate two catalytic subunits forming a tetrameric structure that is conserved from yeast to humans (Taylor et al., 1990; Knighton et al., 1991; Johnson et al., 2001). The binding of Cyclic adenosine 3′,5′ monophosphate (cAMP) to the regulatory subunits of the complex derepresses PKA (Broach, 2012). Therefore, increasing cellular levels of cAMP results in an increase in PKA directed phosphorylation. cAMP levels in the cell are at their highest in the presence of glucose via the combined efforts of adenylate cyclase and phosphodiesterase, which are themselves regulated by signaling proteins that either directly or indirectly respond to glucose levels (Broach, 2012). Activation of PKA in *S. cerevisiae* has been linked to around 90% of glucose induced changes at the transcriptional level (Zaman et al., 2009). Furthermore, the deletion of multiple subunits of the complex results in a strain that is resistant to glucose withdraw induced translational shut-down (Ashe et al., 2000). Interestingly, PKA in exponentially growing cells has been found to physically associate with translation factors, such as Pab1, suggesting that it may promote or inhibit the recognition of bound mRNAs by ribosome initiation factors directly (Gao et al., 2008; Tudisca et al., 2012). Furthermore, PKA may also regulate the abundance of the scaffolding initiation factor 4G, further supporting its role in regulating translational flux (Tudisca et al., 2012).

As it stands with regard to glucose withdraw, removal of the 5′ mRNA cap precedes polysomal collapse with de-adenylation seeming to play a less essential role in *S. cerevisiae* (Ashe et al., 2000). In contrast, the de-adenylating enzyme Ccr4 in *C. neoformans* partly facilitates translational repression in response to carbon starvation (Banerjee et al., 2016). Initiation factors that recognize the 5′ cap or the 3′ poly-A tail to promote translation of the associated mRNA also protect it from the RNA degradation machinery. Therefore, post-translational modifications must exist that either increase the affinity of the decapping enzymes Dcp1/2 or de-adenylating complexes for its substrate, or decrease the affinity of the initiation scaffolding complex affinity for the mRNA. Post-translational modifications made by PKA on these critical factors can favor translation and therefore protect the mRNA from the de-capping machinery during glucose-rich conditions (Figure 3). In Glucose-rich media, an environment that is known to stimulate robust protein biogenesis, PKA is kept in an active state. The removal of glucose rapidly inactivates it, while at the same time, translation is inhibited (Tudisca et al., 2012). Furthering its role in keeping the genetic system in a high translational state, PKA directly phosphorylates Pat1, a crucial component of stress granules (Ramachandran et al., 2011). Stress granules are large mRNA-protein complexes that are believed to be both a site of storage and decay of transcripts. Their formation is tightly correlated with the disassociation of ribosomes from mRNA (Teixeira et al., 2005). In glucose rich conditions, the C terminus of Pat1 is kept phosphorylated, preventing it from forming P bodies. A more recent study found that this C terminus is responsible for observed interaction with Dcp2 and the 5′-3′ exonuclease Xrn1 (Charenton et al., 2017). Is it possible that phosphorylation of this site by PKA prevents the recruitment of the de-capping machinery to the translating mRNAs? It is an attractive hypothesis given that strains lacking Pat1 are also resistant to glucose withdraw induced translational repression (Holmes et al., 2004). Altogether, glucose withdraw seems to initiate a process that promotes a translational system that dis-favors the 5′ mRNA cap. Interestingly, the cap-binding initiation factor 4E in *S. cerevisiae* was found to be dispensable in yeast growing at the glucose poor stationary phase for an extended period (Paz and Choder, 2001). It is possible, then, that these kinases promote a situation where non-canonical forms of translation are favored. It follows that the translation of transcripts that can engage with the translational machinery in a cap-independent manner may be favored.

### Kinase-Regulated Translational Remodeling in Response to Glucose Availability in Pathogenic Fungi

Do these kinases possess a similar function in pathogenic fungi, and are they essential for virulence? As it is for most biological questions, the answer is complicated. In addition to the expected defects in carbon utilization observed in *S. cerevisiae*, the absence of Snf1 in *C. neoformans* also results in an increased sensitivity to nitrosative stress and decreased melanin production at higher temperatures (Hu et al., 2008a). Mice that were challenged intranasally with *snf1*Δ strains survived throughout the experimental condition long after mice challenged with the wild type strain succumbed to infection. In *C. albicans*, Snf1 is believed to be essential for growth as attempts at making homozygous mutants have failed (Petter et al., 1997). Therefore, *C. albicans* seems to have evolved a more substantial dependence on Snf1 than other fungi, and is in contrast to the filamentous fungal pathogen, *Aspergillus nidulans*, where the absence of the Snf1 homolog is not essential for viability. Instead, it regulates the production of hydrolytic enzymes when cellulose is the sole carbon source (Brown et al., 2013). Further microarray-derived data demonstrates that many genes involved in metabolism are dysregulated in the absence of Snf1, mainly in response to glucose removal.

The deletion of both PKA subunits, *TPK1* and *TPK2*, in a single *C. albicans* strain results in severe growth sensitivity (Cao et al., 2017). However, the deletion of only one isoform resulted in a growth sensitivity when in the presence of specific cellular stresses. Unlike *S. cerevisiae*, none of the major components of the Ras/cAMP/PKA pathway by themselves are essential for viability (Jung et al., 2005; Biswas et al., 2007; Zhu et al., 2009). However, *C. albicans* strains lacking PKA activity do not adequately engage in yeast to a hyphal morphological switch and are therefore avirulent (Lo et al., 1997). PKA activity is essential for growth and conidiation in *Aspergillus fumigatus*, resulting in a drastic reduction in virulence in mice (Oliver et al., 2002; Liebmann et al., 2004; Zhao et al., 2006). Virulence is also reduced in *C. neoformans*, where the PKA pathway is required for capsule and melanin production, thereby facilitating mammalian infection. Additional transcriptional analysis of PKA function in *C. neoformans* has been performed demonstrating roles in the induction of cell wall, metabolism, and ribosomal subunit genes (Hu et al., 2007; Banerjee et al., 2016). More recent proteomic data utilizing a PKA inducible and repressible system further corroborates these findings showing 302 proteins whose abundance is regulated by this kinase (Geddes et al., 2016). One of the largest groups found to be regulated were components of the translational machinery, suggesting that PKA signaling influences ribosome composition and availability.

To the best of our knowledge, little work has been done to investigate changes to the translational landscape in response to carbon starvation in any pathogenic fungal system. The gap in research is not surprising as translational regulation (independent of mRNA copy number) has only recently been appreciated as a significant influencer of protein expression (Schwanhausser et al., 2011). Given that the 5′ cap seems to be the main target in glucose withdraw mediated repression in *S. cerevisiae*, it is interesting to find that *C. albicans* can survive (albeit poorly) without enzymes that facilitate mRNA capping (Dunyak et al., 2002). Therefore, translation in these strains must be initiated in a cap-independent manner. Alternative initiation pathways that are irrespective of the methylated cap may aid in the pathogen's ability to facilitate disease by allowing a more robust translational profile when disseminating into poor nutrient tissues (Nicola et al., 2008). Additionally, *C. albicans* may have evolved unique biology in the initiation of translation that has yet to be extensively explored. The most glaring question this finding asks is how a eukaryotic organism can remain viable if the canonical form of translation initiation is inhibited.

## Temperature Dependent Translational Regulation

**Figure 4:**
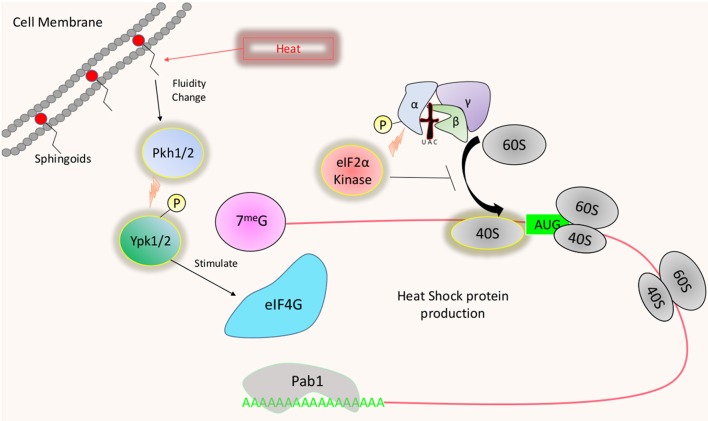
Temperature induced translational regulation: Heat shock results in the transient activation of certain eIF2α kinases, which limits the availability of active ternary complexes. Heat induced changes in cell membrane fluidity may also signal the activation of Ypk1/2 through sphingolipid sensing and activation of Pkh1/2. Activated Ypk1/2 may increase survival during heat stress by increasing the availability of eIF4G.

Drastic increases in temperature cause disruptions in cellular functions stemming from altered membrane dynamics and large scale misfolding of proteins (Verghese et al., 2012). The ability to adjust the proteome accordingly and grow at temperatures exceeding ambient is essential for virulence and restricts many fungi from being pathogenic (Bergman, 1966; Robert and Casadevall, 2009). Several kinases have been identified to play a role in thermotolerance in fungi. However, a vast majority of these studies focused more on the transcriptomic consequences of their activity rather than their impact on translation. As previously mentioned, translation is often drastically suppressed in response to stress at the level of translation initiation. Several studies have examined mild heat shocks of temperature (between 37° and 42°C), more relevant to host temperature adaptation, as well as more robust heat shock up to 50°C. The fission yeast *Schizosaccharomyces pombe* possess three kinases that can phosphorylate eIF2α, Hri1, Hri2, and Gcn2, which target the same conserved serine (Zhan et al., 2004). Although phosphorylation of eIF2α has yet to be examined at mammalian body temperature, robust heat shock (46°C) has been found to induce Hri2 mediated eIF2α phosphorylation (Zhan et al., 2004) (Figure 4). Gcn2 also contributed to this response but was not active until 20–30 min following the heat shock. The importance of eIF2α phosphorylation as it pertains to translational adaptation during heat stress is currently unknown. Similar to the carbon starvation response, Wen et al. demonstrated that stress granules form following heat stress in *S. pombe*, suggesting that ribosome dissociation may be occurring. However, the double knock-out strain lacking both Hri1 and Gcn2 still form stress granules signifying an eIF2α phosphorylation independent form of translational suppression may exist (Martin et al., 2013).

While eIF2α phosphorylation has been documented following both mild and robust heat stress in *S. cerevisiae* (Meier et al., 2006; Grousl et al., 2009), this phosphorylation and its effect on translation are transient. Meier et al. revealed that protein production is reduced ~50% 15 min following mild heat shock, but increases by 150% by 1 h. In agreement with this, polysome profiles show reduced polysomes and increased monosomes indicative of translation initiation arrest at 15 min, and profiles return to normal by 60 min. Furthermore, as demonstrated in *S. pombe*, stress granule formation, and translation arrest following robust heat shock in *S. cerevisiae* are independent of Gcn2p (Grousl et al., 2009).

Though Gcn2 does not appear to play a vital role in translational regulation during heat stress in *S. cerevisiae*, Meier et al. revealed that signaling via sphingoid bases play an essential role in this translational response. The absence of sphingoid bases reduces the immediate translation of heat shock proteins (HSPs) following heat shock in conjunction with prolonged arrest of translation initiation. Ypk1/2 are downstream of Pkh1/2 which are activated by sphingoid bases (Friant et al., 2001; Liu et al., 2005) (Figure 4). While Pkh1 plays a role in the translational induction of HSPs, both Pkh1 and Ypk1 appear to play a role in translation reinitiation following heat shock (Meier et al., 2006). Furthermore, similar to a ypk^ts^ strain shifted to the non-permissive temperature, the lcb1-100 strain (impaired in sphingolipid biosynthesis) displays a reduction in eIF4G following heat shock, further suggesting that sphingoid base signaling via PDK-YPK is a crucial player in translational regulation during heat shock (Gelperin et al., 2002; Meier et al., 2006). Prolonged translational suppression in the lcb-100 strain in response to heat shock was alleviated by the deletion of Eap1, suggesting that the binding interaction between eIF4E and eIF4G may be targets of the stress response. How PDK-YPK signaling may promote virulence in fungi that infect hosts with high body temperatures has yet to be explored in great detail. Deletion of the orthologous PDK1 gene in *C. neoformans* ablates thermotolerance, suggesting that it may be essential for establishing infection in mammals (Chabrier-Rosello et al., 2013). The observed fitness loss in the *pdk1*Δ strains may stem from a defect in mRNA metabolism, as the accelerated decay of the abundant ribosomal protein (RP) transcripts was lost following a shift to host temperature (Bloom and Panepinto, 2014). Given the observed correlation between translation initiation and decay, the absence of mRNA turnover may be the symptom of a defect in translational suppression in response to temperature stress (Chan et al., 2018).

Barraza et al. demonstrated that two of the three partially redundant catalytic subunits of PKA, Tpk2 and Tpk3, are involved in the formation of cytoplasmic granules in response to mild and severe heat shock and are differentially involved in translational regulation (Barraza et al., 2017). While Tpk3 shuttles from the nucleus to cytoplasmic foci following mild (37°C) and severe heat shock (46°C), Tpk2 only forms foci following severe heat shock. In all cases, the formation of these granules is dependent upon translational repression as elongation inhibitor cycloheximide prevents their genesis. Interestingly, while the catalytic activity of Tpk2 was required for its aggregation, Tpk3 aggregation was independent of its catalytic activity. Polysome profiles obtained from individual knockout strains revealed that Tpk2 might promote translation during severe heat stress while Tpk3 promotes translational arrest. The authors suggest that PKA isoforms, therefore, play opposing roles in heat stress.

The topic of ribosome heterogeneity and specialized ribosomes has recently become an attractive avenue of exploration. Though it has yet to be explored in detail, it is conceivable that post-translational modification of ribosomes may permit the translation of specific target mRNAs during stress. Tomioka et al. recently reported that phosphorylation of uS7/Rps5 via Ypk1 regulates small ribosomal subunit biogenesis and translation (Tomioka et al., 2018). The critical residue for this phosphorylation event is S223, and although an S223A uS7 mutant displays a severe growth defect under normal growth conditions coinciding with its defect in translation, it has increased resistance to heat stress compared to the wild type. Authors contributed this thermotolerance to increased production of HSPs and suggested that the S223A mutation may promote selective translation of specific mRNAs. Furthermore, it was proposed that enhanced HSP production and reduced translation in the S223A strain may be protective against ER stress, which is triggered during heat shock. The study suggests then that dephosphorylation of uS7 may be a mechanism to help cope with heat stress, but dephosphorylation of S223 has not been explored. It is tempting to imagine how post-translational modification of ribosomal subunits may differ between thermotolerant fungi and close nonpathogenic relatives. If modifications were found, are they conserved across pathogenic fungi and do they promote translation under higher temperatures? Technology to begin addressing these sort of questions are now becoming available, though in their infancy.

## Summary/Conclusion

It is becoming evident that the once believed passively acting polypeptide machine may be the localization point of multiple cellular stress inputs. All the above mentioned host derived stresses affect the extent and of the translational output of the fungus in diverse ways. Translation suppression in response to glucose withdraw is severe and sustained for an extended period. H_2_O_2_ derived oxidative stress also results in robust translational repression, though this reduction in comparison is gradual over time. Temperature adaptation is unique in that the translational reduction is mild and transient. It is interesting to see that in all three examples, translation is inhibited by different mechanisms, suggesting an underlying fitness benefit. Possibly, stress specific translation inhibition may establish conditions were mRNAs are translationally favored over others based on the path of translational suppression used. We speculate that activation or deactivation of certain kinases stimulates change in the ribosome composition, availability, and post-translational modifications to promote the production of certain mRNAs over others according to the stress input. Therefore, the initial modulation of different kinases can facilitate an organism's “adaptive agility.” Even weakening this response could allow the host immune system time to contend with the infection. The ability to address these questions has only become possible within the past decade with the advent of techniques such as ribosome profiling. Application of these tools to the study fungal gene regulation in response to host derived stresses may reveal the crucial role of translation in establishing a virulent phenotype.

## Author Contributions

JL conceived and wrote parts of the manuscript. AB wrote parts and edited the manuscript. JP conceived and edited the manuscript.

### Conflict of Interest Statement

The authors declare that the research was conducted in the absence of any commercial or financial relationships that could be construed as a potential conflict of interest.
